# Phenotypic Detection of Metallo-Beta-Lactamases in Carbapenem Resistant* Acinetobacter baumannii* Isolated from Pediatric Patients in Pakistan

**DOI:** 10.1155/2016/8603964

**Published:** 2016-03-30

**Authors:** Muneeza Anwar, Hassan Ejaz, Aizza Zafar, Hamdan Hamid

**Affiliations:** ^1^Department of Microbiology, The Children's Hospital and Institute of Child Health Lahore, Pakistan; ^2^Department of Immunology, University of Health Sciences Lahore, Pakistan

## Abstract

Multidrug resistant* A. baumannii* has emerged as an important and problematic human pathogen as it is the causative agent of several types of infections especially in neonates and immunocompromised patients because they have least capacity to fight against infections. Carbapenems are used as last resort antibiotics for treating these infections but currently resistance against carbapenems due to MBL production is on the rise. The objective of this study was to determine the frequency of antibiotic resistance in* A. baumannii* and also to compare the efficacy of combined disk test and double disk synergy test for detection of metallo-beta-lactamases. A total of 112* A. baumannii* were identified from various clinical samples and antibiotic susceptibility profile was determined by Kirby-Bauer Disk Diffusion method. Out of 112, 66 (58.9%) isolates were resistant to both imipenem and meropenem (OXOID). These resistant isolates were tested for carbapenemase production, and 55 (83.3%) were carbapenemase producers by Modified Hodge Test. These isolates were further tested for MBL production by combined disk test and double disk synergy test. Out of 66, 49 isolates were positive by both methods, CDT and DDST, and only one isolate was detected as negative (with kappa value = 0.038). All MBL producing strains showed remarkable resistance to cephalosporins, fluoroquinolones, aminoglycosides, and piperacillin/tazobactam (OXOID). The antibiotic resistance was very high in* A. baumannii* which were isolated from children in Pakistan specially attending a nephrology unit.

## 1. Introduction

*A. baumannii* is a pleomorphic aerobe Gram-negative bacterium. It is a nonmotile, encapsulated, nonlactose fermenting Gram-negative coccobacillus [1]. It is the most important species of genus* Acinetobacter* which has emerged as one of the global multidrug resistant nosocomial pathogens worldwide [2]. Nosocomial infections are a major challenge to patient safety and are the sixth most important cause of death in the United States [3].

*A. baumannii* causes a wide range of infections such as ventilated associated pneumonia, septicaemia, urinary tract infections, wound infections, and meningitis especially in immunocompromised and hospitalized patients [3]. Carbapenems are often used as last resort antibiotics for treating serious nosocomial infections caused by multidrug resistant strains [4]. Although antibiotic resistance is caused by multiple mechanisms, the resistance to carbapenem antibiotics due to carbapenem hydrolysing enzymes is now a worldwide issue [5].

Carbapenem hydrolysing enzymes belong to classes A, B, and D according to molecular Ambler classification and are called carbapenemases. However, the carbapenemases in class B require one or two zinc ions for their full catalytic activity and these enzymes are therefore called MBLs [6]. MBLs are considered to be more crucial than other resistance mechanisms because they can almost hydrolyse all beta-lactam antibiotics [7]. There are no clinically approved MBL inhibitors, making these enzymes a serious threat to human health [8]. MBL encoding genes can be easily disseminated from one bacterium to another through mechanism of horizontal gene transfer [7].

The MBL determinant appears to be widespread in the Indian subcontinent and in India 70–90% of the population may carry MBL producers whereas in Pakistan 27.1% of the population may carry MBL producers and intercontinental propagation of MBL producing species has been acknowledged in Europe, Australia, and Africa [9, 10]. MBLs are now known to be expressed by at least 20 species including most of the members of Enterobacteriaceae family especially* Escherichia coli*,* Klebsiella pneumoniae*,* A. baumannii*,* Bacteroides fragilis*,* Aeromonas hydrophila*,* Stenotrophomonas maltophilia*,* Serratia marcescens*,* and Pseudomonas aeruginosa* [11].

This study was aimed at quantifying the resistance pattern of* A. baumannii* to carbapenems and at detecting the frequency of carbapenemase and MBL producing* A. baumannii* phenotypically, so as to modify therapy and initiate effective infection control measures.

## 2. Materials and Methods

This descriptive cross-sectional study was conducted in the Department of Microbiology, the Children's Hospital and Institute of Child Health Lahore, from July 2014 to December 2014. A total of 112* A. baumannii* were recovered from clinical specimens comprising urine, blood, sputum, pus, body fluids, and tracheal secretions and cultures were performed. Colonies suspected of* A. baumannii* were identified by Gram staining, colony morphology, negative oxidase reaction and API 20E. All the strains which showed resistance to carbapenem were further screened for carbapenemase and MBL production by MHT, DDST, and CDT, respectively.

### 2.1. Antimicrobial Susceptibility

Antibiotic sensitivity testing was performed to determine the sensitivity pattern of test organism by using Kirby-Bauer Disk Diffusion method (Table 1). Different antibiotics of OXOID were tested which included amikacin (AK), gentamicin (CN), cefuroxime (CXM), cefixime (CFM), cefotaxime (CTX), ceftazidime (CAZ), ceftriaxone (CRO), sulbactam/cefoperazone (SCF), ciprofloxacin (CIP), levofloxacin (LEV), moxifloxacin (MOX), meropenem (MEM), imipenem (IPM), colistin sulphate (CT), and piperacillin/tazobactam (TZP).

### 2.2. Modified Hodge Test (MHT)

All the carbapenem resistant strains were subjected to MHT for detection of carbapenemases. The suspension of ATCC* E. coli* 25922 was prepared in comparison to 0.5 McFarland standard in 5 mL of sterile saline using the direct colony suspension. A 4.5 mL of sterile saline was pipetted out into a sterile tube. Then 0.5 mL of the ATCC* E. coli* 25922 suspension was added to 4.5 mL of saline to make a 1 : 10 dilution. Diluted ATCC* E. coli* 25922 was inoculated to a Mueller Hinton (MH) plate containing 70 *μ*g/mL of zinc sulphate and then streaked over the entire plate with the help of sterile cotton swab. A 10 *μ*g meropenem susceptibility disk was placed in the centre of the MH plate. Then the test organism, positive control (*Klebsiella pneumoniae* ATCC BAA-1706) and negative control (*Klebsiella pneumoniae* ATCC BAA-1705), was streaked in a straight line from the edge of the meropenem disk to the edge of the plate and then incubated at 35°C ± 2°C in an ambient air for 16–20 hours. Carbapenemase production was detected by the appearance of the enhanced ATCC* E. coli* 25922 growth along the test organism that revealed a clover-leaf-like indentation which indicated a positive test.

### 2.3. Combined Disk Test (CDT)

CDT was used for the phenotypic detection of MBLs in carbapenem resistant Gram-negative bacteria. An EDTA solution of 0.5 M concentration was prepared by dissolving 46.53 g of disodium EDTA·2H_2_O in 250 mL of distilled water and adjusting it to pH 8.0 by using NaOH [12]. The mixture was sterilized by autoclaving. Two 10 *μ*g imipenem disks were placed on MH agar and 4 *μ*L of an EDTA solution was added to one of them to obtain the desired concentration. The inhibition zones of the imipenem and imipenem-EDTA disks were compared after 16 to 18 hours of incubation at 35°C. In the CDT the increased inhibition zone ≥7 mm with the imipenem-EDTA disk was compared to the imipenem disk alone and was considered as MBL positive [13].

### 2.4. Double Disk Synergy Test (DDST)

The imipenem-EDTA DDST was performed according to a study done by Lee et al. [14]. A 0.1 M solution of EDTA was prepared by dissolving 29.4 g of disodium EDTA·2H_2_O in 250 mL of distilled water and adjusting it to pH 8.0 by using NaOH [12]. The mixture was sterilized by autoclaving. Direct colony suspension of test organism adjusted to match 0.5 McFarland turbidity was prepared and inoculated onto MH agar plate as recommended by the National Committee for Clinical Laboratory Standards (CLSI, 2013) [15]. One blank filter paper disk containing 10 *μ*L EDTA was placed at a distance of 20 mm from the centre of imipenem disk. The inhibition zones of the imipenem and blank disk with EDTA were compared. Enhancement of the zone of inhibition in the area between imipenem and the EDTA disk in comparison with the zone of inhibition on the far side of the drug was interpreted as a positive result [14].

## 3. Results

**Figure 1:**
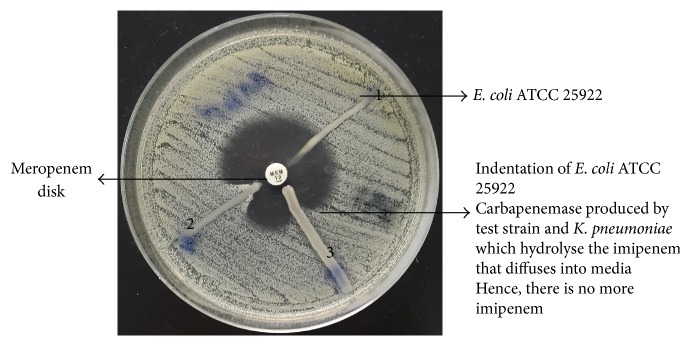
The MHT on a 100 mm MH plate: (1) ATCC BAA-1705* Klebsiella pneumoniae* negative result, (2) ATCC BAA-1706* Klebsiella pneumoniae* positive result, and (3) test strain positive result.

Carbapenem resistance was observed in 66 (58.9%)* A. baumannii* out of 112 clinical isolates by Kirby-Bauer Disk Diffusion method. These carbapenem resistant isolates were tested for carbapenemase production by MHT and 55 (83.3%) were carbapenemase producers (Figure 1).

**Figure 2:**
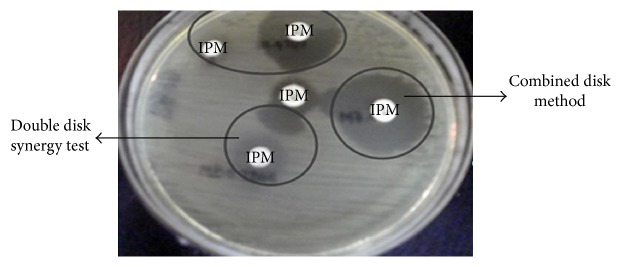
Phenotypic detection of metallo-beta-lactamases by combined disk test and double disk synergy test.

The carbapenem resistant isolates were also tested for MBL production and 63 of these isolates gave positive result by CDT, whereas 51 isolates gave positive result by DDST (Figure 2).

**Figure 3:**
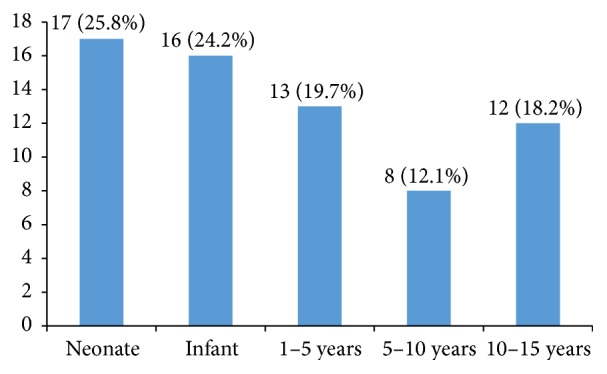
Occurrence of MBL producing* A. baumannii* in different age groups (*n* = 66).

Out of 66 resistant isolates, 49 (74.2%) were collected from male patients. The production of MBLs was seen in majority of the neonates and infants as there were 17 (25.8%) and 16 (24.2%) cases detected, respectively. Less number of MBL strains were isolated from other age groups (Figure 3).

The predominant source of MBL producing strains was from urine (14) (21.2%) followed by blood (12) (18.2%), tracheal secretions (9) (13.6%), cerebrospinal fluid (6) (9.1%), pus (5) (7.6%), peritoneal dialysis catheter (5) (7.6%), wound swab (4)  (6.1%), central venous tips (4) (6.1%), pleural fluid (2) (3.0%), endotracheal tube (2) (3.0%), and sputum (1) (1.5%).

Maximum number of* A. baumannii* was isolated from various wards as follows: nephrology (15) (22.2%), followed by cardiology (12) (18.2%), surgery (9) (13.6%), intensive care unit (7) (10.6%), oncology (7) (10.6%), medical units (7) (10.6), neonatal unit (5) (7.6%), and neurology unit (4) (6.1%).

The overall outcome showed that there were 11 (16.7%) cases of mortalities observed in patients infected with MBL producing* A. baumannii.* Among the other patients 53 (80.3%) were discharged after successful treatment and 2 (3.0%) patients left against medical advice.

## 4. Discussion

Frequency of carbapenem resistance was observed in a study conducted by Hussein et al. (2013) in which (58.26%) isolates were resistant to both meropenem and imipenem. The meropenem resistant strains were screened for carbapenemase production by MHT and 83.3% were identified as carbapenemase producers, which are in agreement with the current study [16]. Kumar et al. (2011) reported that 71% of the isolates were carbapenemase producers by the MHT [17]. This was also in concordance with the results which were reported by Lee et al. (2003) in Korea, where 73% of the isolates were found to be carbapenemase positive by the MHT [14].

The meropenem resistant isolates were further screened for MBL production. 95.4% were positive by CDT and 77.2% were positive by DDST. Similar study conducted by Pandya et al. (2011) showed that 96.30% of isolates were MBL positive by CDT and 81.4% were positive by DDST [18]. The results of present study also correlate with the results of study conducted by Irfan et al. (2008) at Aga Khan University, Karachi, in which 96.6% of the carbapenem resistant strains were MBL producers by CDT [19]. Similar results were obtained with the study conducted by Noori et al. (2014), in which 86.8% of isolates were identified as MBL producers by CDT [7].

Current study suggests that most of the MBL producing* A. baumannii* were isolated from neonates. Begum et al. (2013) performed a study at Quaid-i-Azam, University Islamabad, in which 42.85% neonates had infections caused by MDR* A. baumannii*. There were 74.2% male and 25.8% female patients [20]. A study by Alm-El-Din et al. (2014) reported more males (51.5%) than females (48.8%) [21]. Islahi et al. (2014) also observed a high percentage of nosocomial infections more in males (76.0%) than females (23.9%) [22]. Similar results were seen in a study performed by Peymani et al. (2011) in which 72% were male and 28% were female patients [23].

The most frequent site of infection was urinary tract infections (21.2%) followed by blood stream infections (18.2%) and respiratory infections (13.6%). The results were different from other studies which reported that respiratory tract infections were the common site of infection. Noori et al. (2014) found the highest percentage of MBL producing* A. baumannii* in respiratory tract specimens (52.8%) followed by urine (26.9%) and blood (7.4%) [7]. Another study was conducted by de Carvalho et al. (2013) which described that the most frequent site of isolation was tracheal secretion (56.3%), followed by the catheter tip (16.9%), blood (7%), and urine (7%) [24]. The sites of infection can vary in various patients and this could be associated with the type of interventional procedure.

Sunenshine et al. (2007) performed a retrospective analysis on patients infected with MDR* A. baumannii* in which mortality of infected patients was 26% which is very close to mortality rate of present study which was 16.7% [25]. A similar study was conducted by Kumar et al. (2012) in which the mortality rate due to MDR* A. baumannii* was 16.6% [26].

The frequency of patients infected with MBL producing* A. baumannii* was higher in nephrology ward (22.2%) followed by cardiology (18.2%), surgery (13.6%), and medical intensive care unit (10.6%) which were different from the results of other studies. Mahajan et al. (2011) conducted a prospective study in a tertiary care hospital of Jalandhar and found that the highest percentage belonged to intensive care unit (42.8%) followed by surgery, medicine, and nephrology [27]. According to Muthusamy and Boope (2012), 35% belonged to ICU followed by neurosurgery (19%), neurology (12%), surgery (8%), and nephrology (5%) [3].

Current study depicts that all the MBL producers possess very high resistance to all antimicrobials (beta-lactams, aminoglycosides, and fluoroquinolones) from 71.2% to 100% and also revealed 83.3% resistance to piperacillin/tazobactam. All MBL producing* A. baumannii* showed 15.2% resistance to colistin sulphate and 18.2% to sulbactam/cefoperazone. The present study showed that colistin sulphate and sulbactam/cefoperazone were the most effective drugs for the treatment of MDR* A. baumannii*. Islahi et al. (2014) determined a resistance pattern of* Acinetobacter* species in hospitalized patients. Their study showed that 84.7% of isolates were resistant to amikacin, 86.9% to gentamicin, 89.1% to ceftriaxone and cefotaxime, 86.4% to ciprofloxacin, 10.6% to colistin sulphate, and 32.7% to sulbactam/cefoperazone [22]. Similar results were seen in a study performed by Jaggi et al. (2012) according to which 90.3% were resistant to amikacin, 85.8% to gentamicin, 92.1% to ceftazidime, 89.7% to piperacillin, 91.6% to ciprofloxacin, 87.6% to levofloxacin, and 1.2% to colistin [28]. Noori et al. (2014) reported that 95.4% of isolates were resistant to ceftazidime, 40.7% to gentamicin, 80.6% to amikacin, 92.6% to ciprofloxacin, 95.4% to piperacillin/tazobactam, and 1.8% to colistin sulphate [7].

## 5. Conclusion

The present study demonstrates the presence of high level of multiantibiotic resistance among carbapenem resistant* Acinetobacter baumannii* isolates from pediatric nephrology patients as assessed in 2014 in Pakistan. Comparison of multiple phenotypic assays for the detection of metallo-beta-lactamases in bacteria indicates that the combined disk test provides the highest rate of positive tests.

## Figures and Tables

**Table 1:** Antimicrobial sensitivity of MBL producing *A. baumannii*.

Antibiotics	Resistant (%)	Intermediate sensitive (%)	Sensitive (%)
Cefuroxime	66 (100.0)	0 (0.0)	0 (0.0)
Cefixime	66 (100.0)	0 (0.0)	0 (0.0)
Meropenem	66 (100.0)	0 (0.0)	0 (0.0)
Imipenem	66 (100.0)	0 (0.0)	0 (0.0)
Ceftriaxone	65 (98.5)	1 (1.5)	0 (0.0)
Cefotaxime	65 (98.5)	1 (1.5)	0 (0.0)
Ceftazidime	64 (97.0)	0 (0.0)	2 (3.0)
Ciprofloxacin	61 (92.4)	0 (0.0)	5 (7.6)
Levofloxacin	57 (86.4)	1 (1.5)	8 (12.1)
Moxifloxacin	57 (86.4)	1 (1.5)	8 (12.1)
Piperacillin/tazobactam	55 (83.3)	5 (7.6)	6 (9.1)
Amikacin	55 (83.3)	4 (6.1)	7 (10.6)
Gentamicin	47 (71.2)	0 (0.0)	19 (28.8)
Sulbactam/cefoperazone	12 (18.2)	18 (27.3)	36 (54.5)
Colistin sulphate	10 (15.2)	0 (0.0)	56 (84.8)

## Additional Points

The study depicts the presence of high level of antibiotic resistance in MBL producing* Acinetobacter baumannii* specially related to nephrology patients in children of Pakistan.

## References

[1] Bérézin E. B., Towner K. J. (1996). *Acinetobacter* spp. as nosocomial pathogens: microbiological, clinical, and epidemiological features. *Clinical Microbiology Reviews*.

[2] Singla P., Sikka R., Deep A., Chaudhary U. (2013). Phenotypic detection and prevalence of MBLs in carbapenem resistant isolates of *Acinetobacter* species at a tertiary care hospital in north India. *International Journal of Pharma and Bio Sciences*.

[3] Muthusamy D., Boope A. (2012). Phenotypic methods for the detection of various beta-lactamases in carbapenem resistant isolates of *Acinetobacter baumanii* at a tertiary care hospital in South India. *Journal of Clinical and Diagnostic Research*.

[4] Shobha K. L., Lenka P. R., Sharma M. K., Ramachandra L., Bairy I. (2009). Metallo-*β*-lactamase production among *Pseudomonas* species and *Acinetobacter* species in costal Karnataka. *Journal of Clinical and Diagnostic Research*.

[5] Kabbaj H., Seffar M., Belefquih B. (2013). Prevalence of metallo-*β*-lactamases producing *Acinetobacter baumannii* in a Moroccan hospital. *ISRN Infectious Diseases*.

[6] Schlesinger S. R., Lahousse M. J., Foster T. O., Kim S.-K. (2011). Metallo-*β*-lactamases and aptamer-based inhibition. *Pharmaceuticals*.

[7] Noori M., Karimi A., Fallah F. (2014). High prevalence of metallo-beta-lactamase producing *Acinetobacter baumannii* isolated from two hospitals of Tehran, Iran. *Archives of Pediatric Infectious Diseases*.

[8] King D., Strynadka N. (2011). Crystal structure of New Delhi metallo-*β*-lactamase reveals molecular basis for antibiotic resistance. *Protein Science*.

[9] Perry J. D., Naqvi S. H., Mirza I. A. (2011). Prevalence of faecal carriage of *Enterobacteriaceae* with NDM-1 carbapenemase at military hospitals in Pakistan, and evaluation of two chromogenic media. *Journal of Antimicrobial Chemotherapy*.

[10] D'Andrea M. M., Venturelli C., Giani T. (2011). Persistent carriage and infection by multidrug-resistant *Escherichia coli* ST405 producing NDM-1 carbapenemase: report on the first Italian cases. *Journal of Clinical Microbiology*.

[11] Liu Z., Li W., Wang J. (2013). Identification and characterization of the first *Escherichia coli* strain carrying NDM-1 gene in China. *PLoS ONE*.

[12] Prajapati S. B., Vegad M. M., Mehta S. J., Kikani K. M., Kamothi M. N., Pandya J. M. (2011). An evaluation of two different phenotypic methods for detection of metallo-*β*-lactamase producing *Pseudomonas* isolates. *Journal of Cell & Tissue Research*.

[13] Yong D., Lee K., Yum J. H., Shin H. B., Rossolini G. M., Chong Y. (2002). Imipenem-EDTA disk method for differentiation of metallo-*β*-lactamase-producing clinical isolates of *Pseudomonas* spp. and *Acinetobacter* spp.. *Journal of Clinical Microbiology*.

[14] Lee K., Lim Y. S., Yong D., Yum J. H., Chong Y. (2003). Evaluation of the Hodge test and the imipenem-EDTA double-disk synergy test for differentiating metallo-*β*-lactamase-producing isolates of *Pseudomonas* spp. and *Acinetobacter* spp.. *Journal of Clinical Microbiology*.

[15] Clinical and Laboratory Standards Institute (CLSI) (2013). Performance standards for antimicrobial susceptibility testing; twenty-third informational supplement.

[16] Hussein H. N., Al-Mathkhury J. F. H., Sabbah A. M. (2013). Imipenem resistant *Acinetobacter baumannii* isolated from patients and hospitals environment in Baghdad. *Iraqi Journal of Science*.

[17] Kumar A. V., Pillai V. S., Dinesh K., Karim S. (2011). The phenotypic detection of carbapenemase in meropenem resistant acinetobacter calcoaceticus-baumannii complex in a tertiary care hospital in South India. *Journal of Clinical and Diagnostic Research*.

[18] Pandya P. N., Prajapati B. S., Mehta J. S., Kikani M. K., Joshi J. P. (2011). Evaluation of various methods for detection of MBL (MBL) production in Gram negative bacilli. *International Journal of Biological and Medical Research*.

[19] Irfan S., Zafar A., Guhar D., Ahsan T., Hasan R. (2008). Metallo-*β*-lactamase-producing clinical isolates of *Acinetobacter* species and *Pseudomonas aeruginosa* from intensive care unit patients of a tertiary care hospital. *Indian Journal of Medical Microbiology*.

[20] Begum S., Hasan F., Hussain S., Shah A. A. (2013). Prevalence of multi drug resistant *Acinetobacter baumannii* in the clinical samples from Tertiary Care Hospital in Islamabad, Pakistan. *Pakistan Journal of Medical Sciences*.

[21] Alm-El-Din A. R., El-Bassat H., El-Bedewy M., El-Mohamady H. (2014). Prevalence of metallo-*β*-lactamases producers among carbapenem-resistant *Acinetobacter baumannii* strains isolated from diabetic foot ulcers. *African Journal of Microbiology Research*.

[22] Islahi S., Ahmad F., Khare V. (2014). Prevalence and resistance pattern of *Acinetobacter* species in hospitalized patients in a tertiary care centre. *Journal of Evolution of Medical and Dental Sciences*.

[23] Peymani A., Nahaei M.-R., Farajnia S. (2011). High prevalence of metallo-beta-lactamase-producing *Acinetobacter baumannii* in a teaching hospital in Tabriz, Iran. *Japanese Journal of Infectious Diseases*.

[24] de Carvalho R. M. L., Marques S. G., Gonçalves L. H. B., Abreu A. G., Monteiro S. G., Gonçalves A. G. (2013). Phenotypic detection of metallo-*β*-lactamases in *Pseudomonas aeruginosa* and *Acinetobacter baumannii* isolated from hospitalized patients in São Luis, State of Maranhão, Brazil. *Revista da Sociedade Brasileira de Medicina Tropical*.

[25] Sunenshine R. H., Wright M.-O., Maragakis L. L. (2007). Multidrug-resistant *Acinetobacter* infection mortality rate and length of hospitalization. *Emerging Infectious Diseases*.

[26] Kumar S. H., De A. S., Baveja S. M., Gore M. A. (2012). Prevalence and risk factors of metallo *β*-lactamase producing *Pseudomonas aeruginosa* and *Acinetobacter* species in burns and surgical wards in a tertiary care hospital. *Journal of Laboratory Physicians*.

[27] Mahajan G., Sheemar S., Chopra S., Kaur J., Chowdhary D., Makhija S. K. (2011). Carbapenem resistance and phenotypic detection of carbapenemases in clinical isolates of *Acinetobacter baumannii*. *Indian Journal of Medical Sciences*.

[28] Jaggi N., Sissodia P., Sharma L. (2012). *Acinetobacter baumannii* isolates in a tertiary care hospital, antimicrobial resistance and clinical significance. *Journal of Microbiology and Infectious Diseases*.

